# Fecal microbiota transplantation and peri-stoma microbiome management in patients with intestinal stomas: from mechanisms to clinical translation

**DOI:** 10.3389/fonc.2026.1913974

**Published:** 2026-09-22

**Authors:** Qiujun Huang, Huifei Xiang, Qiaoyun Liu, Yating Zhang, Junying Zou, Zhanqun Gao, Ling Yang, Hui Zeng, Jian Liu

**Affiliations:** 1 Department of Gastrointestinal Surgery, General Hospital of Hunan University of Medicine, Huaihua, Hunan, China; 2 Department of Anesthesiology, General Hospital of Hunan University of Medicine, Huaihua, Hunan, China; 3 Department of Gynecology, General Hospital of Hunan University of Medicine, Huaihua, Hunan, China; 4 Department of Cardiology, General Hospital of Hunan University of Medicine, Huaihua, Hunan, China; 5 General Hospital of Hunan University of Medicine, Huaihua, Hunan, China; 6 Department of Physical Examination Center, Qinhuangdao First Hospital, Qinhuangdao, Hebei, China

**Keywords:** anastomotic leak, bacteriophage, diversion colitis, engineered microbiome, fecal microbiota transplantation, gut microbiota, perioperative management, stoma

## Abstract

**Background:**

Fecal diversion-induced dysbiosis in the defunctioned intestinal segment represents a common pathogenic basis for diversion colitis, anastomotic leakage, and long-term metabolic sequelae in patients with permanent stomas. This review proposes the novel concept of “Peri-stoma Microbiome Management (PSMM)” and critically evaluates the clinical evidence for fecal microbiota transplantation (FMT), defined microbial consortia, bacteriophages, and postbiotics. It is important to emphasize that PSMM is a proposed conceptual framework; most of its components have not been prospectively validated in stoma patients.

**Main body:**

The core pathological process involves a dramatic reduction in butyrate-producing obligate anaerobes, depriving colonocytes of their primary energy substrate. In experimental models, butyrate depletion impairs mitochondrial function and autophagic flux, with subsequent NLRP3 inflammasome activation; whether this pathway operates identically in the defunctioned human colon remains to be directly investigated. Collagenolytic pathobionts such as *Enterococcus faecalis* may expand under antibiotic pressure and secrete matrix metalloproteinase-9 that degrades anastomotic extracellular matrix. However, anastomotic leakage is a multifactorial complication, involving tissue ischemia, tension, patient comorbidities, and microbial factors. The PSMM framework encompasses four phases: (I) pre-stoma creation microbial pre-habilitation and risk prediction; (II) microbiota monitoring and intervention during stoma maintenance; (III) pre-reversal targeted decolonization using bacteriophages and engineered microbiota reconstruction; and (IV) long-term metabolic maintenance for permanent stoma carriers. Bacteriophages show promise for precision decolonization but face significant challenges, including bacterial resistance, narrow host range, manufacturing complexity, and regulatory barriers. Multi-omics monitoring remains aspirational due to high costs, long turnaround times, and lack of clinical integration.

**Conclusion:**

The PSMM framework provides a structured conceptual approach for integrating microbiome-targeted strategies into the care of patients with intestinal stomas. However, most interventions remain investigational and require validation in adequately powered randomized trials.

## Introduction

### Clinical definitions and context

Before introducing the conceptual framework that is the focus of this review, it is necessary to define several clinical entities central to the discussion.

Diversion colitis is a non-specific inflammatory condition that develops in the defunctioned segment of bowel following fecal stream diversion. It is characterized endoscopically by mucosal erythema, friability, edema, and nodularity, and histologically by crypt distortion, lymphoid hyperplasia, and mucosal atrophy. The condition affects 30–60% of patients with a stoma and is primarily attributable to the absence of luminal short-chain fatty acids (SCFAs), particularly butyrate, which normally serve as the primary energy substrate for colonocytes (1–3).

High-output stoma (HOS) is defined as an ileostomy output exceeding 1,500 mL per 24 hours. It occurs in approximately 10–16% of patients following ileostomy creation and can lead to severe dehydration, electrolyte disturbances (particularly hyponatremia and hypokalemia), acute kidney injury (AKI), and malnutrition requiring long-term parenteral nutrition (4, 5). The condition is associated with significant morbidity, frequent hospital readmissions, and impaired quality of life.

Temporary versus permanent stomas present distinct clinical challenges. Temporary stomas, typically created to protect a distal anastomosis and reversed within 2–6 months, expose patients to a finite period of diversion-related dysbiosis. The defunctioned colon undergoes mucosal atrophy during this period, and its recovery after restoration of continuity requires 4–12 weeks (6). Permanent stomas, in contrast, result in lifelong absence of colonic fermentation, with accumulating evidence suggesting an association with long-term metabolic consequences including increased risks of metabolic syndrome, osteoporosis, and cardiovascular disease (7–9).

### Rationale for microbiome-directed management

Worldwide, approximately 300,000–500,000 new intestinal stomas are created annually, predominately as a consequence of colorectal cancer, inflammatory bowel disease (IBD), diverticulitis, trauma, and congenital anomalies (1, 2). Although a protective ileostomy substantially reduces the re-operation rate for symptomatic anastomotic leakage after low anterior resection, the stoma itself is far from benign (4). Diversion colitis affects 30–60% of stoma patients (5, 6, 10); high-output ileostomy (>1,500 mL/24h) occurs in 10–16% of cases (7); and the anastomotic leakage rate after stoma reversal remains 5–15% (8) (**Figure 1**).

**Figure 1:**
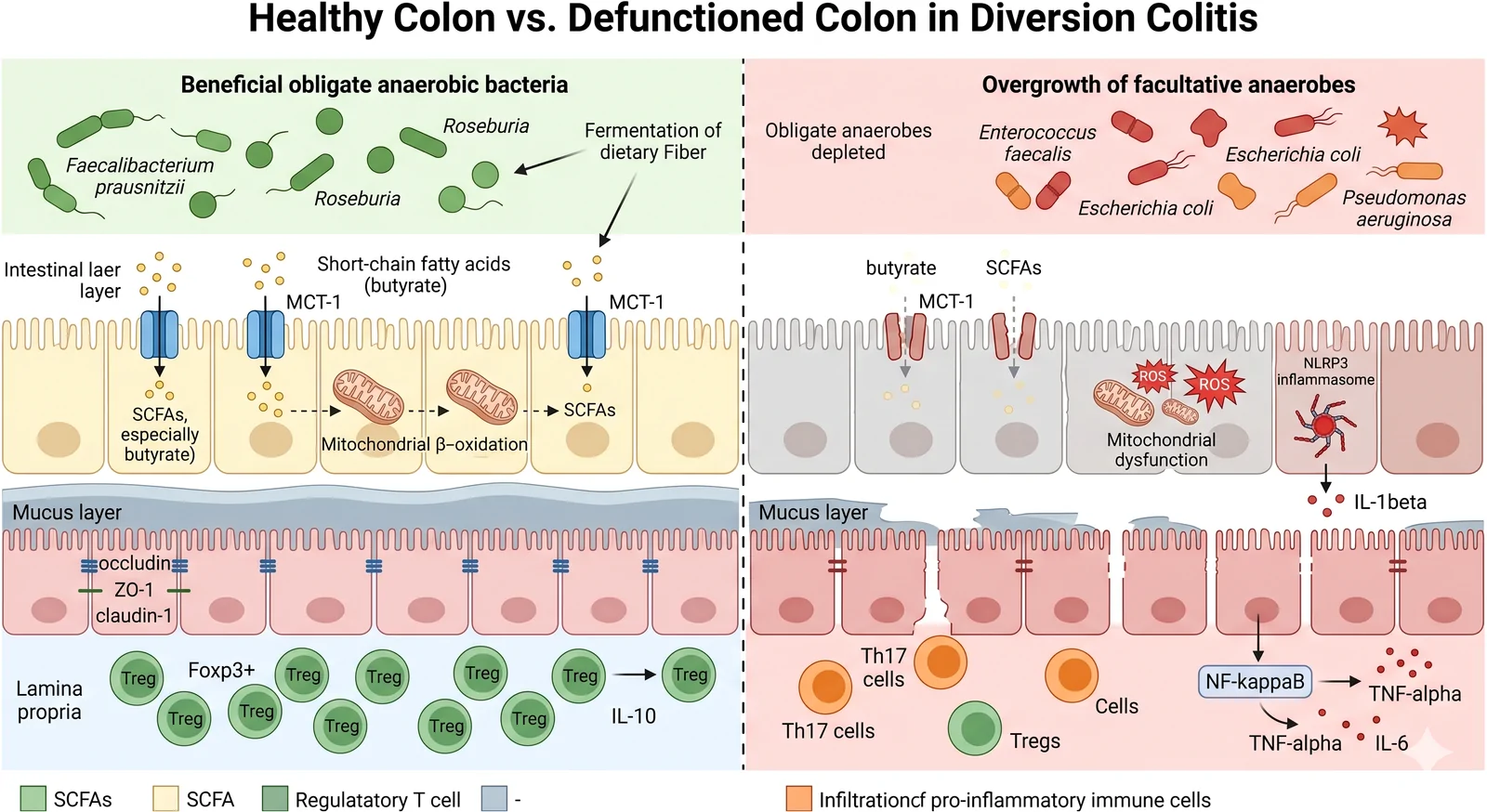
Healthy vs. defunctioned colon: the top panel illustrates obligate anaerobes producing butyrate, fueling colonocyte β-oxidation and maintaining tight junctions and Treg-cell-mediated tolerance; the bottom panel shows diversion-induced loss of butyrate, mitochondrial stress, NLRP3 inflammasome activation, junctional breakdown, and Th17-skewed inflammation.

The gut microbiota is an active “microbial organ” maintaining mucosal homeostasis through metabolic cross-feeding, immune education, and colonization resistance (11–13). Following fecal diversion, the microbiota-host network rapidly collapses due to the abrupt cessation of dietary fiber substrates. Two principal pathological axes link stoma complications: the “butyrate starvation–mucosal atrophy axis” and the “collagenolytic pathobiont expansion–anastomotic leak axis” (14–16). FMT, by restoring the normal microbiota-metabolism-immune network, has the potential to reverse these processes.

FMT has achieved cure rates of 80–85% after a single infusion for recurrent *Clostridioides difficile* infection (rCDI) (17–19). Its perioperative application has expanded rapidly (20–22), and next-generation therapeutics—defined microbial consortia and lytic bacteriophage preparations—have entered clinical trials (23–26). Because no existing review systematically integrates microbiome management across the entire stoma lifecycle, this article introduces the framework of “Peri-stoma Microbiome Management (PSMM).”

## The PSMM framework: a proposed conceptual model

It is important to emphasize at the outset that the Peri-stoma Microbiome Management (PSMM) framework described herein is the authors’ proposed conceptual model. Most of its components have not been prospectively validated in stoma patients, and their clinical adoption should await evidence from adequately powered randomized trials. The framework is intended to provide a structured approach for organizing current evidence and identifying priority areas for future investigation.

Phase I: Pre-stoma creation. Preoperative assessment of the gut microbiome may identify patients with elevated abundance of collagenase-producing organisms. In such patients, preoperative autologous FMT or dietary pre-habilitation could be considered to enhance microbial resilience, though this approach warrants prospective evaluation (27, 28). Preoperative metagenomic screening for collagenolytic pathobionts represents a potential strategy for risk stratification, but prospective validation in surgical cohorts is lacking (29, 30).

Phase II: Stoma maintenance. For patients who develop diversion colitis, FMT delivered via the efferent limb of a loop stoma has shown promise in small case series with reported clinical response rates of approximately 70% (31, 32). However, these data derive from case reports and small uncontrolled series; larger prospective studies are required before this can be considered a standard therapeutic option. Routine periodic microbiome monitoring may eventually prove useful for early detection of dysbiosis, but cost-effectiveness, optimal sampling frequency, and choice of biomarkers remain undefined (**Figure 2**).

**Figure 2:**
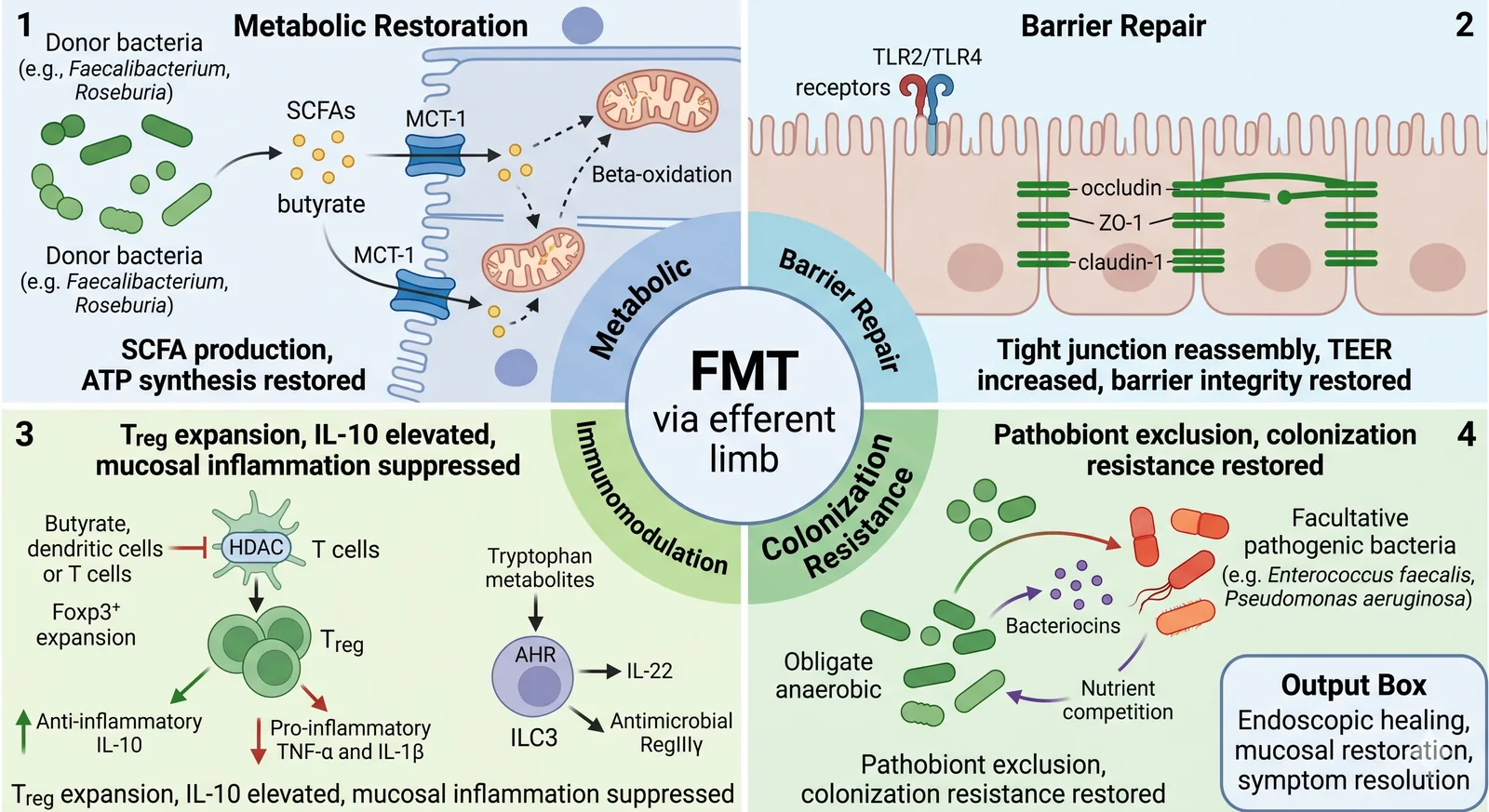
Multi-target mechanisms of FMT in diversion colitis: the central node “Efferent-limb FMT” radiates to four quadrants—(1) metabolic restoration (butyrate replenishment, mitochondrial β-oxidation), (2) barrier repair (TLR-mediated tight junction reassembly), (3) immunomodulation (HDAC inhibition, Treg expansion, IL-10 upregulation), and (4) colonization resistance (nutritional exclusion and bacteriocin-mediated suppression of pathobionts).

Phase III: Pre-reversal optimization. Pre-reversal FMT or defined microbial consortia represents an investigational strategy to optimize anastomotic healing. Preliminary data suggesting reduced AL rates are encouraging but derive from small trials with methodological limitations (33, 34). Phage therapy for targeted decolonization of *E. faecalis* prior to stoma reversal is a novel concept supported by preclinical evidence in rodent models (35); no human data currently exist.

Phase IV: Long-term management. Annual metabolomic surveillance of permanent stoma carriers is hypothesized to enable early detection of metabolic complications, but the evidence linking specific metabolite profiles to clinical outcomes in this population is indirect. Biomarker-guided intervention algorithms may eventually facilitate personalized risk stratification, but their development requires large-scale prospective cohort studies.

### Stoma-related dysbiosis: dynamic evolution and molecular mechanisms

The microbial composition of ileostomy effluent deviates profoundly from that of healthy feces. Ohigashi et al. (2013) first documented a dramatic drop in SCFA concentrations, with butyrate reaching only 5–10% of normal fecal levels (36). Obligate anaerobes plummeted from 40–60% relative abundance in healthy stool to <10%, while facultative anaerobes and antibiotic resistance genes were enriched (37, 38). Metabolomics showed accumulation of primary bile acids and near-complete absence of secondary bile acids (39), which may aggravate secretory diarrhea (40).

Mucosal biopsies from the defunctioned colon revealed a shift from obligate anaerobes to facultative aerotolerants (41, 42). In experimental models, butyrate depletion impairs mitochondrial function and autophagic flux, with subsequent NLRP3 inflammasome activation; whether this pathway operates identically in the defunctioned human colon remains to be investigated (43, 44). Downregulation of tight junction proteins increases epithelial permeability, allowing microbial translocation and NF-κB-driven inflammation (45, 46). Foxp3^+^ Treg cells decline while Th17 cells expand (47).

Colostomy preserves a portion of functional colon, resulting in milder dysbiosis. Ileostomy patients may require more aggressive microbiota reconstruction strategies (48, 49). Even with short-term diversion, mucosal atrophy requires 4–12 weeks to recover (50, 51). Dysbiosis before reversal is positively associated with AL risk (52). Permanent stoma patients face higher rates of metabolic syndrome, osteoporosis, and elevated TMAO levels (53–56), mediated by disrupted SCFA and bile acid signaling (57–59).

### Anastomotic leakage: a multifactorial complication

Anastomotic leakage (AL) following gastrointestinal surgery is a multifactorial complication. While the microbial hypothesis has gained considerable attention, AL rarely results from a single causative factor. Established non-microbial contributors include: tissue perfusion and ischemia; mechanical factors such as excessive tension and suboptimal stapling technique; patient-related factors including diabetes, obesity, smoking, corticosteroid use, malnutrition, and advanced age; and operative factors such as surgeon experience and the level of the anastomosis (20, 21, 60).

While collagenase-producing organisms such as *E. faecalis* have been mechanistically implicated in AL through experimental models (23), it is unlikely that any single microbial species is universally causative. Rather, dysbiosis likely interacts with host factors to increase susceptibility in a subset of patients.

### Beyond *Enterococcus faecalis*: the polymicrobial nature of AL

While *E. faecalis* has been the most intensively studied collagenase-producing organism, other pathogens also contribute. **Proteus mirabilis** produces both collagenase and gelatinase and has been isolated from leaking anastomoses in experimental models (25). Collagenase-producing anaerobes such as *Clostridium perfringens* and certain *Bacteroides* species can degrade extracellular matrix components under low-oxygen conditions. Importantly, multi-species biofilms may exhibit synergistic collagenolytic activity—a concept of “community pathogenicity” implying that targeting a single pathogen may be insufficient if polymicrobial interactions are involved (**Figure 3**).

**Figure 3:**
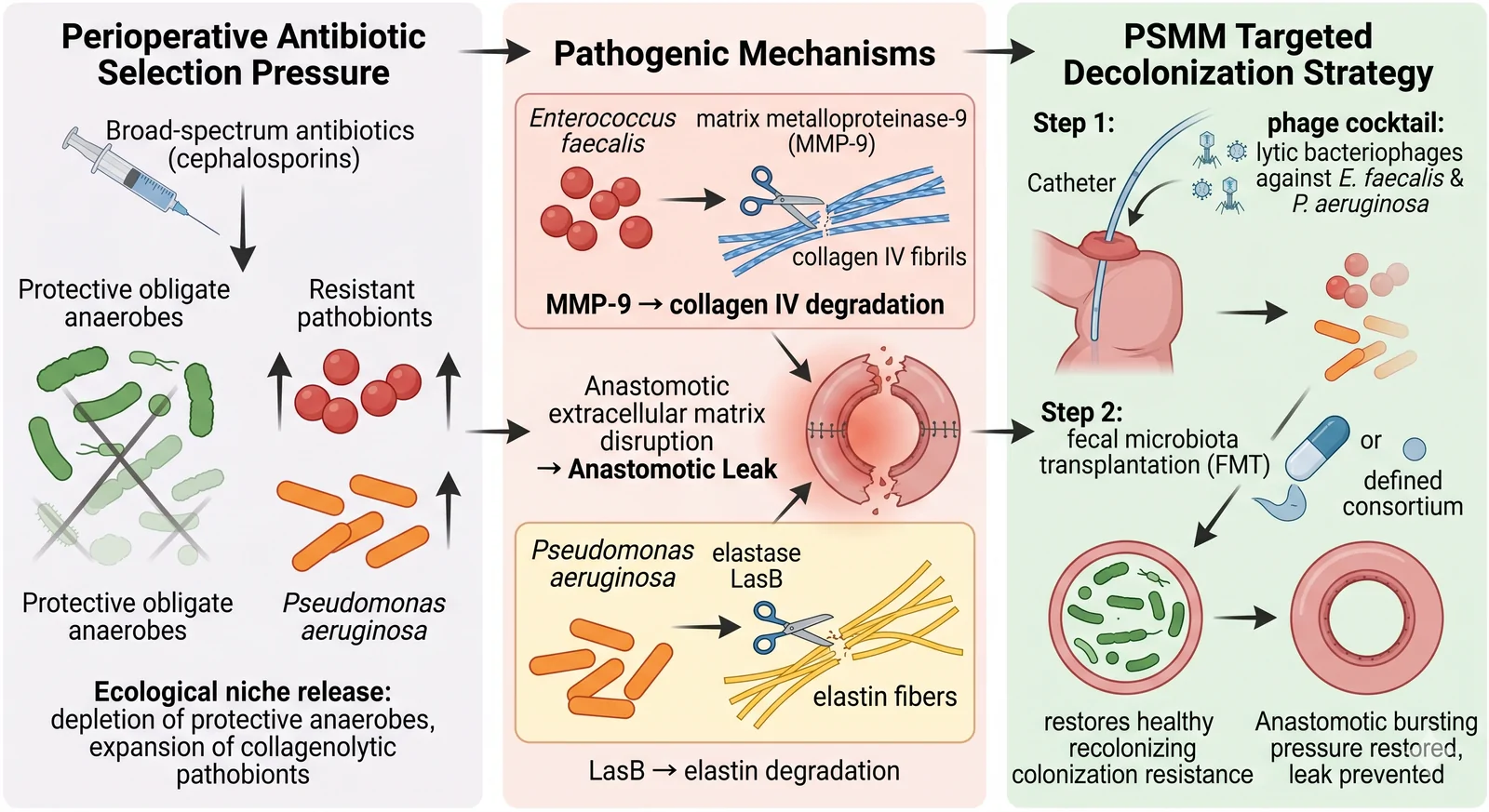
Microbial pathogenesis of anastomotic leak and targeted decolonization. Left: perioperative antibiotics deplete protective anaerobes, enabling collagenolytic *E. faecalis* and elastolytic *P. aeruginosa* to expand via niche release. Center: MMP-9 and LasB degrade collagen IV and elastin, respectively, weakening the anastomotic extracellular matrix. Right: PSMM-guided sequential therapy—phage cocktail administered via the efferent limb specifically eliminates pathobionts, followed by FMT or defined consortium to restore colonization resistance and anastomotic integrity.

### Mechanistic pathways: evidence levels and limitations

Throughout this review, we have distinguished the strength of evidence supporting various mechanistic claims (**Table 1**).

**Table 1:** Evidence levels for key mechanistic pathways.

Pathway	*In vitro*	Animal model	Human observational	RCT
Butyrate depletion → NHE3 downregulation → Na^+^ malabsorption	++	++	+	−
H_2_S excess → CFTR hyperactivation → Cl^-^ hypersecretion	++	+	±	−
*E. faecalis* collagenase → anastomotic ECM degradation	++	++	+	±
IS/PCS tubular toxicity → oxidative stress → fibrosis	++	++	++	−
FMT restoration of SCFA → epithelial repair	+	++	+	±
Phage decolonization → reduced AL	+	+	−	−

++ Strong evidence; + Moderate evidence; ± Preliminary/indirect; − No direct evidence.

### PSMM phase I: pre-stoma creation

Mechanical bowel preparation and oral broad-spectrum antibiotics reduce fecal α-diversity by 50–70%, creating a niche for antibiotic-resistant facultative anaerobes (61, 62). In a randomized trial, preoperative *Lactobacillus rhamnosus* GG reduced postoperative infections (63). For high-risk patients, autologous FMT could be considered to competitively exclude collagenolytic pathobionts, but warrants prospective evaluation (27, 28).

### PsSMM phase II: stoma maintenance

FMT has shown promise for diversion colitis. In small case series, colonoscopic FMT achieved endoscopic remission in 80% of patients (31), and efferent-limb FMT improved endoscopic scores (32, 64). A 2024 systematic review estimated a 72% response rate, but evidence quality is low (65). Donor butyrate-producer abundance correlates with improvement (66, 67).

High-output ileostomy is linked to bile acid malabsorption. A case report documented FMT-related reduction in stoma output (68, 69); FMT restores secondary bile acid synthesis (69, 70). However, evidence remains preliminary (71). Efferent-limb catheter instillation is endoscopy-free and home-based, with 85% compliance in a feasibility study (72).

### PSMM phase III: pre-reversal optimization

Shogan et al. demonstrated *E. faecalis* collagenase-mediated degradation of anastomotic collagen IV (73). Several RCTs have explored pre-reversal FMT (33, 34, 74, 75), with pooled data showing a trend toward reduced AL (RR 0.42) but low certainty of evidence. The concept of microbiota-sparing antibiotic stewardship—using narrow-spectrum anti-*E. faecalis* agents—is mechanistically appealing but lacks prospective validation (76, 77).

### Bacteriophage therapy: opportunities and challenges

Lytic bacteriophages enable precision decolonization as shown in preclinical models (35), but important challenges remain (78):

• Resistance development through receptor mutation, CRISPR-Cas, and restriction-modification systems. Phage cocktails may mitigate but not eliminate this risk.
• Narrow host range necessitating patient-specific susceptibility testing.
• Manufacturing complexity for biological products, requiring rigorous quality control.
• Regulatory barriers: existing frameworks are not designed for personalized phage cocktails.

### PSMM phase IV: long-term management

Annual microbiota–metabolite monitoring may eventually detect early metabolic complications, but clinical utility remains unproven. The proposed risk-stratified intervention algorithm requires validation in prospective cohorts (53, 54). After stoma reversal, faster recovery of butyrate-producers is associated with lower LARS scores, but evidence is observational (79) (**Figure 4**).

**Figure 4:**
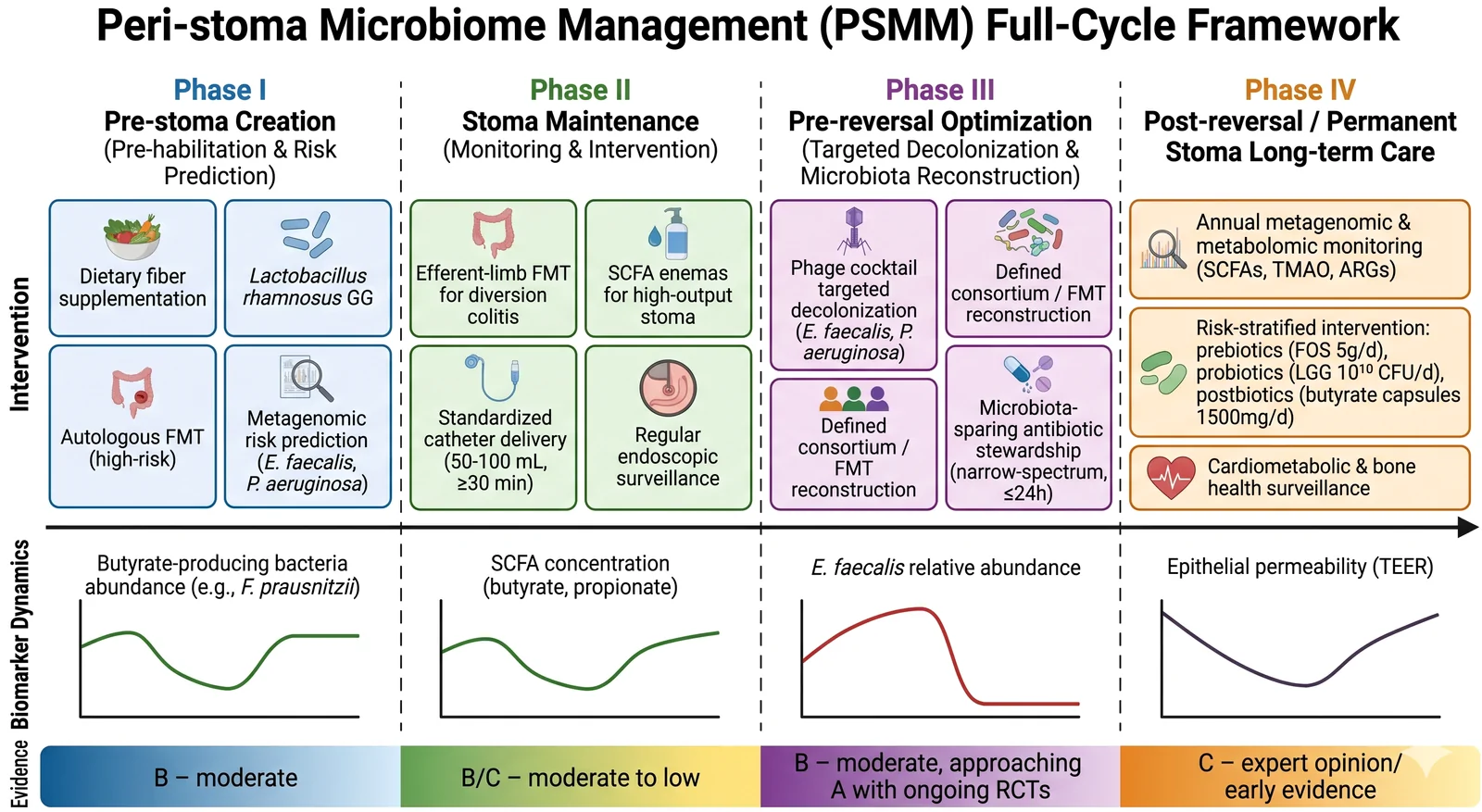
The PSMM full-cycle framework. A horizontal timeline divides the stoma lifecycle into four phases (I: pre-stoma creation; II: stoma maintenance; III: pre-reversal optimization; IV: post-reversal/permanent stoma long-term care). Above the timeline, key interventions are listed for each phase (e.g., autologous FMT, metagenomic risk prediction, phage decolonization, defined consortia, annual multi-omics monitoring). Below the timeline, dynamic trends of biomarkers (butyrate-producer abundance, SCFA concentration, E. faecalis load, epithelial permeability) are shown as line graphs or heatmaps. A bottom evidence-level bar uses a color gradient to indicate current evidence grades **(A–C)** for interventions in each phase.

### Next-generation microbiome therapeutics

Defined consortia and phage cocktails have shown advantages in preclinical models (80–85) but remain investigational. Postbiotics provide safe alternatives for immunocompromised patients (86–88) (**Figure 5**).

**Figure 5:**
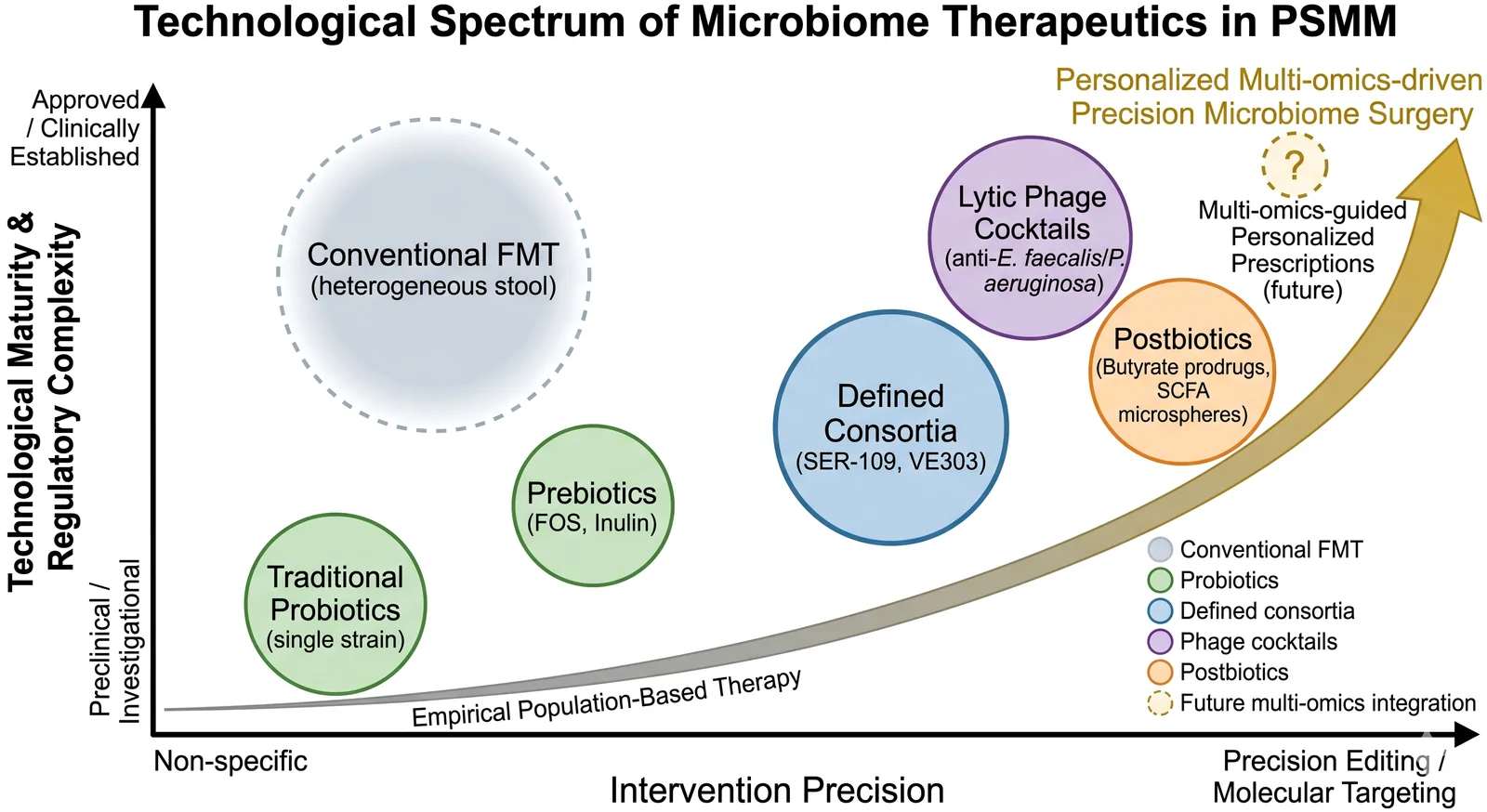
Technological spectrum of microbiome therapeutics in PSMM. A two-dimensional plot positions conventional FMT (low precision, moderate maturity), defined consortia (high precision, moderate maturity), phage cocktails (high precision, early maturity), postbiotics (variable precision, maturing), and future multi-omics-guided prescriptions (highest precision, emerging) along axes of “Intervention Precision” and “Technological Maturity & Regulatory Complexity”. A gradient arrow illustrates the transition from empirical population-based therapy to personalized precision microbiome surgery.

### Multi-omics integration: practical barriers

Practical barriers currently limit clinical feasibility:

• Cost: USD 200–500 per sample for sequencing and metabolomics.
• Turnaround time: 4–8 weeks, incompatible with real-time decisions.
• Computational infrastructure: requires specialized expertise and high-performance computing.
• Clinical feasibility: requires dedicated personnel, standardized protocols, and EHR integration.

## Conclusions

The PSMM framework provides a structured conceptual approach for organizing microbiome-targeted strategies in stoma patients. However, most proposed interventions remain investigational and require validation in adequately powered randomized trials. Anastomotic leakage is multifactorial, with microbiota representing one contributing factor among many. Future research should prioritize prospective validation of the interventions discussed, with careful attention to practical barriers, regulatory pathways, and the polymicrobial nature of anastomotic failure.
